# Molecular Mechanisms of the Genetic Predisposition to Acute Megakaryoblastic Leukemia in Infants With Down Syndrome

**DOI:** 10.3389/fonc.2021.636633

**Published:** 2021-03-11

**Authors:** Juliane Grimm, Dirk Heckl, Jan-Henning Klusmann

**Affiliations:** ^1^Pediatric Hematology and Oncology, Martin Luther University Halle-Wittenberg, Halle, Germany; ^2^Department of Internal Medicine IV, Oncology/Hematology, Martin Luther University Halle-Wittenberg, Halle, Germany

**Keywords:** ML–DS, transient myeloproliferative disorder of Down syndrome, TAM, genetic predisposition, Trisomy 21 (Down syndrome), acute myeloid leukemia, acute megakaryoblastic leukemia

## Abstract

Individuals with Down syndrome are genetically predisposed to developing acute megakaryoblastic leukemia. This myeloid leukemia associated with Down syndrome (ML–DS) demonstrates a model of step-wise leukemogenesis with perturbed hematopoiesis already presenting *in utero*, facilitating the acquisition of additional driver mutations such as truncating *GATA1* variants, which are pathognomonic to the disease. Consequently, the affected individuals suffer from a transient abnormal myelopoiesis (TAM)—a pre-leukemic state preceding the progression to ML–DS. In our review, we focus on the molecular mechanisms of the different steps of clonal evolution in Down syndrome leukemogenesis, and aim to provide a comprehensive view on the complex interplay between gene dosage imbalances, *GATA1* mutations and somatic mutations affecting JAK-STAT signaling, the cohesin complex and epigenetic regulators.

## Background: Leukemic Predisposition in Down Syndrome

Trisomy 21 (T21), which results in the development of Down syndrome (DS), is the most frequent numeric chromosomal aberration with an incidence of approximately one case in 1,000 births (1). Besides many complications resulting from T21—such as craniofacial dysmorphia, cognitive deficits, and congenital heart defects—DS individuals are known to have a 150-fold increased risk of suffering from myeloid leukemia within their first years of life (2). In contrast, the risk of developing solid malignancies is significantly decreased in DS individuals (3, 4), arguing against DS being a general cancer predisposition.

Myeloid leukemia associated with DS (ML–DS) phenotypically reflects acute megakaryoblastic leukemia (AMKL) observed in patients without DS. However, unlike non-DS-AMKL, patients with ML–DS harbor an excellent prognosis (5).

ML–DS displays a model of step-wise leukemogenesis. T21 already perturbs hematopoiesis *in utero*, causing pronounced megakaryocytic and erythroid lineage commitment and proliferation (6–10). As early as during fetal liver hematopoiesis, mutations in the hematopoietic master regulator GATA1 are acquired, leading to the exclusive expression of a N-terminal truncated isoform (GATA1s) and loss of the full length transcription factor (11–20). *GATA1*s mutations are indispensable for ML–DS leukemogenesis, as they are found in almost all ML–DS individuals (12). The consequence of the *GATA1*s mutations are uncontrolled expansion of fetal megakaryocytic cells and perturbed terminal erythroid differentiation (6, 21–23). This leads to a disease called transient abnormal hematopoiesis (TAM), which is usually diagnosed within the first week after birth and occurs in about 10–30% of DS individuals (24, 25). TAM is a pre-leukemic state as the course of disease is usually self-limiting within the first months of life (25). However, TAM clones can persist, acquire additional somatic driver mutations, and finally give rise to ML–DS.

To date, it is not fully understood why some patients with TAM progress to ML–DS and others do not. However, large sequencing studies of TAM and ML–DS samples shed new light on the molecular landscape of ML–DS (26–28) and give insight into the transformative character of many somatic mutations (27). On the other hand, the role of “third hit” mutations that also occur in TAM patients who do not develop ML–DS needs to be established (27). In addition, the molecular mechanisms of T21-driven genetic predisposition to myeloid leukemia have been extensively studied, but still need further characterization.

In this review article, we summarize what we have learned from studies on the molecular background of T21-driven genetic predisposition to myeloid leukemia and from analyzing the consecutive steps during DS leukemogenesis, and how this has increased our knowledge of the pathogenesis of leukemia beyond ML–DS. We also include the most recent insights into the molecular landscape of ML–DS and outline what are the remaining open questions to fully understand the role of T21 in leukemia.

## Impact of T21 on Hematopoiesis

Previous studies have shown that T21 severely affects hematopoiesis *in utero*, even in the absence of additional mutations (*e.g. GATA1* mutations). Due to difficulties in the accessibility of primary material, induced pluripotent stem cells (iPSCs) have been used to model hematopoiesis during embryogenesis and fetal development. In an iPSC model of primitive hematopoiesis derived from yolk sac progenitors, T21 and euploid controls formed comparable proportions of hematopoietic progenitors. However, the T21 cells were biased towards erythropoiesis, producing erythroblasts and normoblasts at higher percentages, while neutrophils were reduced compared to euploid samples (29). This iPSC model—mirroring primitive hematopoiesis—did not show increased megakaryopoiesis, suggesting that embryonic hematopoiesis is unlikely to be the origin of ML–DS development (29). In contrast, other studies investigating the impact of T21 on definitive fetal liver hematopoiesis demonstrated enhanced megakaryopoiesis in addition to the increased erythroid differentiation of T21 iPSC and primary T21 fetal liver hematopoietic stem cells (HSCs) (6–10). This pronounced megakaryocyte–erythroid differentiation was accompanied by an increased frequency and clonogenicity, not only of HSCs but also of megakaryocytic–erythroid progenitors (MEPs) (8, 9, 23). As a result of the enlarged MEP compartment during fetal liver hematopoiesis, the proportion of common myeloid progenitors (CMP) and granulocytic-monocytic progenitors (GMP) was reduced (7, 10). Additionally, germline T21 led to a differentiation block in B cell development (9).

The observed differences during distinct steps of *in utero* hematopoiesis point toward a developmental stage-specific effect of T21 on hematopoietic stem and progenitor cells (HSPCs), which might be even increased by the shift of hematopoiesis to the fetal liver and a consequent change of the microenvironment.

Comparable to the observations in human primary material, murine Down syndrome models consistently display perturbed hematopoiesis with expansion of HSPCs and the megakaryocytic department (30–32). Ts65Dn mice—a model with partial trisomy of murine chromosome 16 which contains about two third of the homologues on the human chromosome 21—even develop a myeloproliferative disease with dysplastic megakaryopoiesis (33). However, this phenotype is only observed in aged mice, and the relevance of these findings for our understanding of ML–DS—a fetal disease—remains unclear.

## Molecular Mechanisms of Perturbed Hematopoiesis in T21 Individuals

**Figure 1:**
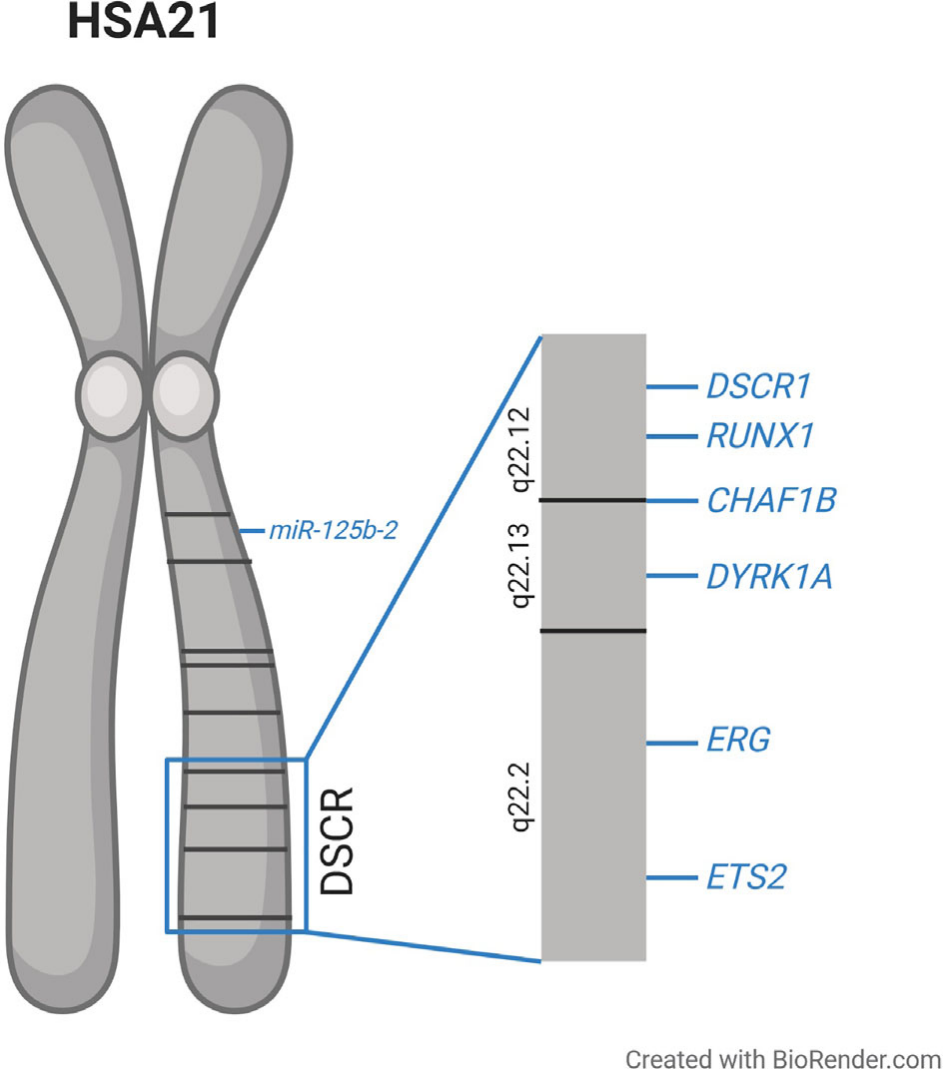
Schematic overview of human chromosome 21 (HSA21) and the proposed Down syndrome critical region (DSCR), along with the location of the genes discussed in this article which are suggested to be involved in the dysregulated hematopoiesis observed in individuals with trisomy 21.

The molecular basis of T21-driven perturbation of hematopoiesis has been intensively studied, but remains to be fully understood. Early genotype–phenotype correlations—especially of cases with partial T21—suggested that a circumscribed region on chromosome 21 is essential for the majority of DS phenotypes, which resulted in the concept of a Down syndrome critical region (DSCR) (34–38). Initially the DSCR was mapped to the bands 21q22.2–21q22.3 including ~6 Mb and 25–50 genes (35–38). However, with an increasing number of studies, it became clear that there might be different critical regions on chromosome 21 for distinct phenotypes, rather than one region being responsible for all phenotypes (39–42). Concerning ML–DS, an around 4 Mb segment was identified that seems to be essential for T21-driven leukemogenesis (**Figure 1**). This segment comprises ~20 genes including *RUNX1*, *ERG*, and *ETS2* (**Figure 1**), which play a pivotal role in hematopoietic differentiation (23, 42). To further understand the molecular mechanisms of T21 altered hematopoiesis, multiple studies analyzed the expression of those and other genes in T21 HSPCs. These investigations consistently demonstrated only slight increases in gene expression not exceeding a two-fold upregulation, which is at least partially explained by increased gene dosages resulting from T21 (7–9, 29).

This supports the concept of only mildly elevated expression of a plurality of genes having extensive effects on downstream targets and regulatory circuits and thereby cooperating to perturb hematopoiesis in individuals with T21. In the following, we focus on specific genes on chromosome 21, which seem to play pivotal roles in the pathogenesis of T21-driven leukemogenesis.

### RUNX Family Transcription Factor 1

RUNX1 is an indispensable transcription factor for hematopoiesis, and forms the core binding factor transcription complex together with its subunit CBF*β* (43). Complete *Runx1* deficiency is incompatible with life, since *Runx1^−/−^* murine embryos die around E12.5 in the absence of fetal liver hematopoiesis (44, 45). There are at least three different RUNX1 isoforms resulting from alternative splicing: RUNX1a, RUNX1b, and RUNX1c. While RUNX1b and RUNX1c contain the DNA binding Runt homologous domain and the transactivation domain, RUNX1a lacks the latter (46). Previously, imbalance of the *RUNX1* isoforms was implicated in leukemogenesis, with RUNX1a exhibiting a pro-leukemic effect *in vivo* (47).

Additionally, *RUNX1* is a translocation partner contributing to numerous fusion oncogenes in AML, of which *RUNX1-ETO* resulting from t(8;21) is the most common, presenting in 12% of cases with *de novo* AML (48).

RUNX1 seems to be essential for lineage programming, as its upregulation precedes megakaryocytic differentiation, while it becomes downregulated upon erythroid lineage commitment (49). In this context, RUNX1 cooperates with GATA1 in the promoter activation of megakaryocytic genes through direct protein-protein interaction (49).

It was shown that perturbation of hematopoiesis in T21 individuals is at least partially executed *via* upregulation of *RUNX1* expression. In a T21 iPSC model, increased *RUNX1* gene dosage caused an expansion of the HSPC pool, especially in early fetal hematopoiesis (23). In contrast, in the Ts65Dn murine DS model, restoring disomy of the *Runx1* locus reduced the number of megakaryocytic colonies but did not completely abrogate the myeloproliferative disease observed in elderly mice, pointing towards the cooperation of multiple genes in DS leukemogenesis (33).

### ETS Transcription Factor ERG and ETS Proto-Oncogene 2

The ETS transcription factor ERG is a proto-oncogene that is essential for HSC maintenance and megakaryocytic differentiation (50–54). Along with its transcription factor family member ETS2, *ERG* was shown to be upregulated in AML with complex karyotypes involving chromosome 21 and in patients with AMKL with or without DS (55, 56). Overexpression of *ERG* as well as *ETS2* caused a switch from erythroid to megakaryocytic differentiation in K562 cells (50, 56). In the regulation of megakaryopoiesis, ETS transcription factors might cooperate with GATA1, since many genes essential for megakaryopoiesis harbor GATA along with ETS binding motifs in their promoters (57, 58). Ectopic expression of *ERG* or *ETS2* together with *GATA1* knock-down induced immortalization of fetal liver cells, as demonstrated in serial replating assays (53).

*In vivo*, increased expression of *ERG* during fetal hematopoiesis led to an expansion of MEPs while GMPs were reduced, comparable to changes observed in T21 individuals (59). Ectopic expression of *ERG* in *Gata1* mutated cells further amplified megakaryocytic differentiation while terminal erythroid differentiation was blocked. Additionally, these *ERG*/*Gata1s* mice demonstrated liver fibrosis and postnatal transient expansion of megakaryocytic progenitor cells, demonstrating that interaction between increased *ERG* expression and *Gata1s* is sufficient to cause a disease with key features of TAM in a murine model (59).

### Dual Specificity Tyrosine Phosphorylation Regulated Kinase 1A

DYRK1A was shown to be a key regulator of calcineurin/NFAT signaling, which is involved in many developmental processes, such as organogenesis, neuronal growth and T cell function (60–63). Moreover, *Nfatc2^−/−^* and *Nfatc4^−/−^* double knockout mice develop typical craniofacial features comparable to the changes observed in human DS (64).

Upon cellular Ca^2+^ intake, calcineurin is activated leading to the dephosphorylation of NFATc proteins. Consequently, NFATc is transported into the nucleus where it activates transcription together with other binding partners (65). NFATc is exported to the cytoplasm upon rephosphorylation, which is executed by glycogen synthase kinase 3 (GSK3) and DYRK1A (60, 64, 66, 67). Hence, *DYRK1A* and *DSCR1* encode inhibitors of calcineurin/NFAT signaling and are both located in the DSCR on chromosome 21 (**Figure 1**). It was shown that a 1.5-fold increase of *DYRK1A* and *DSCR1* expression drastically reduced calcineurin/NFAT pathway activity (64, 68).

Increased *Dyrk1a* gene dosage was previously linked to megakaryocytic leukemogenesis in the Ts1Rhr Down syndrome mouse model (32). Overexpression of *Dyrk1a* in the bone marrow of these mice led to megakaryocytic expansion, which was even more pronounced in the presence of the *Gata1s* mutation. In addition, inhibition of the calcineurin/NFAT pathway by treatment with ciclosporin A in T21 and euploid samples suggested that increased *Dyrk1a* expression causes megakaryocytic expansion at least partially by downregulating the calcineurin/NFAT signaling (32).

### Chromatin Assembly Factor 1 Subunit B

*CHAF1B* encodes the subunit of the chromatin assembly factor 1 complex (CAF1), which is essential for nucleosome assembly during S phase (69, 70). It is located in the DSCR on chromosome 21 (**Figure 1**) and was shown to be overexpressed in ML–DS compared to non-DS-AMKL (32). Additionally, *CHAF1B* overexpression promotes murine megakaryopoiesis (32). In *KMT2A*-rearranged AML, *CHAF1B* overexpression induces a differentiation block and promotes HSPC proliferation (71). Taken together these findings suggest that increased *CHAF1B* gene dosage due to T21 might contribute to the megakaryocytic differentiation block observed in TAM and ML–DS.

### miR-125b-2

MicroRNAs are 21 to 23 nucleotide long non-coding RNAs, which execute post-transcriptional regulation of gene expression by binding to the 3′UTR of their target mRNA and leading to mRNA degradation (72). *MiR-125b-2* is encoded on chromosome 21 (**Figure 1**) and its overexpression in MEPs and megakaryocytic progenitors was shown to enhance self-renewal capacity and proliferation (73). When overexpressed in HSPCs, *miR-125b-2* caused a myeloid differentiation block. The expansion of megakaryocytic cells induced by *miR-125b-2* was even more pronounced in *Gata1s* fetal liver cells, pointing towards synergistic properties in DS leukemogenesis (73). It remains open, whether the other members of the *miR-99a~125b-2* tricistron (*miR-99a* and *let-7c*) on chromosome 21 further enhance or inhibit the oncogenic effects of *miR-125b* in concert with GATA1s (74).

## Development of TAM: The Role of GATA1s in DS Leukemogenesis

The origin of TAM *in utero* marks the second step in DS leukemogenesis. Acquiring a *GATA1* mutation—leading to the loss of full length GATA1 expression—in T21 fetal HSPCs is both sufficient and essential for TAM pathogenesis.

### Natural History of *GATA1s* Mutations in TAM and ML–DS

Exclusive translation of the short isoform of GATA1 is found in over 90% of TAM and ML–DS cases (12). Thus, we can infer that *GATA1s* mutations occur very early during leukemogenesis, most likely during fetal hematopoiesis (11, 15, 17, 18). To date, it is not clear whether the presence of certain *GATA1s* mutations increases the risk of progression to ML–DS. While Alford et al. showed that the type of *GATA1s* mutation is not predictive for transformation from TAM to ML-DS (12), Kanezaki et al. demonstrated a correlation between the mutation type and *GATA1*s expression levels and that low *GATA1s* expression in TAM patients is significantly associated with a higher risk of progression to ML-DS (75). In most cases, the *GATA1s* mutation identified in the TAM sample is also detectable after progression to ML–DS (13, 16), suggesting clonal evolution from TAM to ML-DS. This is even the case if the *GATA1s* clone was not detectable during complete remission. However, new *GATA1*s clones can also arise and contribute to the dominant clone in ML-DS (26). Hence, the dominant *GATA1s* clone can differ between TAM and ML–DS, indicating that evolution from minor TAM clones is a mechanism of ML–DS development (11, 26). *GATA1s* mutations are hardly found in euploid individuals who develop AMKL, underlining the specificity for DS leukemogenesis (11, 76).

### GATA1 in Normal Hematopoiesis

Altogether there are six GATA genes, all of which encode for DNA binding proteins that play a pivotal role in transcriptional regulation (77, 78). The six members of the GATA family all harbor two zinc fingers as their common structure. While the C-terminal zinc finger binds DNA *via* recognition of the GATA motif, the N-terminal zinc finger interacts with important cofactors such as FOG1 (79–82).

*GATA1* is located on the X chromosome and encodes an essential transcription factor for hematopoiesis—especially for the erythroid and megakaryocytic lineages, but also for the development of eosinophil and basophil granulocytes and mast cells (83).

To ensure proper megakaryocytic and erythroid differentiation, the tight transcriptional regulation of *GATA1* and its family member *GATA2* is crucial, and is also referred to as the “GATA switch”. While the expression of *GATA2* is mandatory for the self-renewal capacity of HSPCs, high GATA1 levels are needed for the transition to MEPs and the subsequent differentiation of the megakaryocyte–erythroid lineage (84, 85). This switch between GATA transcription factor expression is realized through the direct transcriptional regulation of GATA1 by GATA2 and *vice versa*, as well as through epigenetic mechanisms, such as DNA methylation (85, 86). It was already shown that, as a consequence of the loss of full length *Gata1*, the “GATA switch” is impaired, causing perturbation of erythropoiesis (87).

Loss of GATA1 expression leads to an erythroid differentiation block and apoptosis of erythroid precursors (23, 88–93). *GATA1* knock-out in murine embryonic stem cells results in embryonic lethality between days 10.5 and 11.5 due to anemia (88). Consistently, *GATA1* knock-out drastically impairs megakaryocytic maturation, resulting in reduced platelet counts. However, immature megakaryocytes undergo excessive proliferation (27, 94, 95).

These findings underline the pivotal role of full length GATA1 for megakaryocyte and erythroid differentiation.

### Functional Consequences of *GATA1* Mutations on Hematopoiesis

In line with the crucial role of GATA1 in physiological hematopoiesis, germline *GATA1* mutations are associated with hereditary thrombocytopenia, dyserythropoietic anemia, and Diamond-Blackfan anemia. However, the majority of germline *GATA1* mutations do not increase the probability of developing leukemia in the absence of T21 (96–100). To date, there is only one published case of a newborn who was diagnosed with TAM at birth, who had an N-terminal *GATA1* mutation but no T21 or any copy-number alterations conformable with T21. However, the identified *GATA1* mutation was a large deletion resulting in the loss of the entire N-terminal zinc finger and parts of the transactivation domain of the transcription factor (101).

In contrast, *GATA1* mutations associated with TAM and ML–DS are typically small insertions or deletions or point mutations in exon 2, which lead to the introduction of a premature stop codon or loss of the adjacent splice site (11–20). As a consequence, only the short isoform GATA1s (~ 40 kD)—which lacks the first 83 amino acids, including the N-terminal transactivation domain (12–17, 19)—is translated from a start codon in exon 3. As a result, GATA1s contains both zinc finger domains, but possesses reduced transactivation potential compared to the full length protein (19).

Additionally, GATA1s shows perturbed binding and activation of important erythroid genes, while its transcriptional activation of megakaryocytic and myeloid target genes is comparable to full length GATA1 (6). Other studies suggest that altered gene expression in the presence of GATA1s might also result from the loss of transcriptional repression at certain GATA1 target genes (102, 103). In general, GATA1s induced changes in transcriptional regulation might be caused by disturbed binding with co-factors, such as RB1 and E2F (104–106). Of note, in fetal liver cells, the expression of the *GATA1* V205G mutant, which is unable to interact with FOG1, did not lead to megakaryocytic hyperproliferation but prevented cells from undergoing terminal differentiation, while GATA1s rescued the megakaryocytic differentiation block in GATA1 deficient cells but sustained uncontrolled expansion (107).

In addition, changes in gene regulation by GATA1s and the resulting hematopoietic alterations also seem to be developmental stage-specific, comparable to perturbations of hematopoiesis caused by T21. In iPSC models of early hematopoiesis derived from yolk sac progenitors and fetal hematopoiesis, GATA1s caused impaired erythropoiesis, even in the presence of T21, thus overriding the pronounced erythroid differentiation caused by T21 (6, 22). On the contrary, GATA1s enhanced the proliferation of dysplastic megakaryocytes—a phenotype which is independent from, but which becomes accelerated in, a T21 background during fetal hematopoiesis (21–23). When *GATA1* mutations were introduced into neonatal HSPCs using a CRISPR-Cas9-system, increased proliferation of erythroid precursors was observed. However, the accumulation of immature erythroid cells was only transient and applying the same method to adult HSPC caused only mildly increased proliferation of the erythroid lineage (21). These results, obtained from *in vitro* studies using primary human material, are in line with data from *GATA1s* knock-in mice demonstrating transient reduction of erythropoiesis and aberrant hyperproliferation of megakaryocytic progenitors during fetal hematopoiesis, but normal hematopoietic differentiation in adult mice (103).

Two recent studies in murine embryonic stem cells (ES) and human T21 iPSCs narrowed down the search for the cellular origin of TAM to a population of immature megakaryocytic progenitors characterized by high CD41 expression (108, 109). During step-wise hematopoiesis *in vitro*, these cells showed delayed and aberrant megakaryocytic differentiation, reduced erythroid differentiation and gave rise to an increased number of myeloid cells upon *GATA1s* expression (108, 109).

### IGF Signaling as Mechanisms of Developmental-Stage Specific Effects of *GATA1s* Mutations

Perturbation of hematopoiesis caused by *GATA1s* mutations as well as T21 show strong dependency on the stage of development. The fact that *GATA1s* knock-in mice display normal hematopoiesis in adult life (103) along with the self-limiting course of TAM in the majority of patients (25) suggests an important role of the fetal liver microenvironment, since hematopoiesis is shifted from the fetal liver to the bone marrow after birth. It was previously shown that fetal liver stromal cells secrete a variety of cofactors supporting the expansion of HSCs, *e.g.* IGF2 (110, 111).

In contrast to equivalent adult cells, fetal megakaryocytic progenitors depend on the IGF/IGFR1/mTOR pathway for proliferation and differentiation, which is constantly active in the fetal liver microenvironment (104). In the presence of continuous IGF/IGFR1 signaling, megakaryocytic expansion needs to be tightly controlled, which is at least partially realized by regulation of the E2F transcription factor. While E2F is activated by the IGF/IGFR1/mTOR cascade, direct interaction with GATA1 inhibits E2F and consequently its downstream targets, *e.g. MYC* (104, 105). However, GATA1s shows reduced binding to the E2F factors and the inhibitory RB1 protein, resulting in an overactivation of E2F target genes and uncontrolled expansion of megakaryocytic progenitors (104). Consistently, another study demonstrated insufficient repression of the E2F transcription network and MYC as reasons for increased proliferation of eosinophil precursors after ectopic *GATA1s* expression in fetal HSPC (112).

These data suggest that the hyperproliferative phenotype in the presence of *GATA1s* mutations results from the overactivity of pro-proliferative genes as a consequence of ineffective suppression of the E2F transcription factor, and deregulated IGF signaling, which might be even further pronounced in a T21 genetic background and fetal liver microenvironment.

### Synergy Between T21 and GATA1s in TAM Pathogenesis

Given that a *GATA1s* mutation in the T21 genetic background is mandatory for the development of TAM, cooperative effects between both aberrations have to be assumed. As previously discussed, T21 causes an expansion of MEPs during fetal liver hematopoiesis (8, 9, 23). This enlarged pool of cells with increased proliferative capacity might be especially susceptible to the acquisition of *GATA1s* mutations. GATA1s leads to hyperproliferation of the megakaryocytic lineage – an effect that is increased in the presence of T21, as previously shown (21–23). Thus, the hypothesis of a positive selection for randomly emerging *GATA1s* mutations in the megakaryocyte–erythroid compartment during T21 fetal liver hematopoiesis seems rational.

Further supporting the idea of cooperation between T21 and GATA1s, it was shown that GATA1s expression is elevated in T21 iPSCs compared to euploid cells (23). In the T21 background, increased gene dosage of *RUNX1*, *ERG*, and *ETS2* upregulate GATA1s expression, which itself further enhances transcription of *RUNX1*, *ERG*, and *ETS2* (23, 87). Besides a direct interaction between *Gata1s* and *Runx1*, another mechanism of increased *Runx1* expression in *Gata1s* erythroid cells is the reduction of the repressive H3K27me3 mark and higher chromatin accessibility at the *Runx1* locus (87). As a consequence of this, gene levels rise two to three-fold compared to euploid cells, leading to the hyperproliferation of aberrant megakaryocytic cells (23).

GATA1 was implicated along with RUNX1, ERG, FLI-1, TAL1, LYL1, and LMO2 to be part of a heptad of transcription factors which cooperatively control gene transcription via DNA and protein-protein interaction upon differentiation of HSPCs (113). Increased gene dosages of *RUNX1*, *ERG*, and *ETS2* together with exclusive GATA1s expression might lead to disruption of this regulatory network, resulting in the pronounced megakaryocytic and impaired erythroid differentiation observed in TAM.

**Figure 2:**
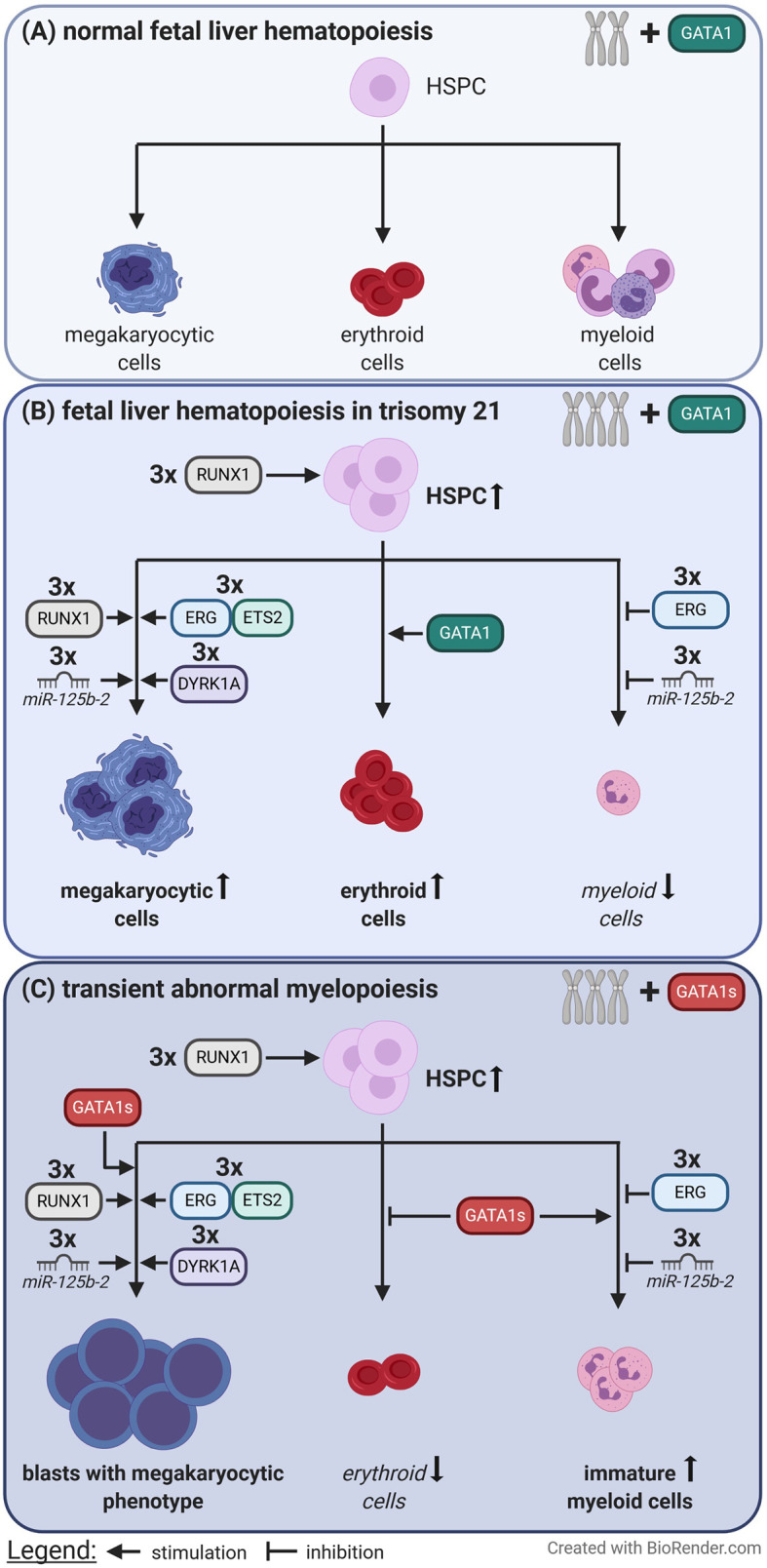
Perturbation of fetal liver hematopoiesis caused by trisomy 21 and loss of full length GATA1. **(A)** In euploid individuals the balanced gene dosages of chromosome 21 and the presence of full length GATA1 contribute to normal fetal liver hematopoiesis. **(B)** In contrast trisomy 21 causes expansion of hematopoietic stem and progenitor cells (HSPC), megakaryocytic and erythroid cells via increased gene dosages. **(C)** When an additional *GATA1* mutation, which leads to the expression of only the short isoform of GATA1 (GATA1s), is acquired immortalized megakaryocytic blasts rapidly expand at the expense of erythropoiesis. This is known as transient abnormal myelopoiesis which typically originates during fetal liver hematopoiesis.

However, further studies are needed to completely understand the difficult interplay between gene dosage changes due to T21 and the disruption of regulatory circuits, and to determine how these alterations translate into leukemogenesis (**Figure 2**).

## Clinical Characteristics and Management of TAM

About 10% of neonates with DS experience TAM—characterized by the clonal proliferation of myeloid blasts with a megakaryoblastic or erythroblastic phenotype, which are detected in peripheral blood (24, 114, 115). Morphologically, TAM blasts cannot be distinguished from ML-DS blasts. Typically, the abundance of TAM blasts in the peripheral blood is not accompanied by a high bone marrow infiltration (116, 117).

TAM is usually diagnosed within the first week after birth, underlining that the disease originates *in utero*. Although stringent diagnostic criteria for TAM do not exist, the diagnosis is usually confirmed by the presence of typical TAM blasts in peripheral blood and the presence of T21 and a *GATA1s* mutation (11, 18). Still, defining TAM is complicated by the fact that the percentage of TAM blasts in the peripheral blood highly varies during the course of the disease, and that individuals without clinical signs of TAM might also harbor *GATA1s* mutations and thus be at risk for developing ML–DS (11).

The clinical presentation ranges widely, from asymptomatic children to fatal cases resulting in early death due to organ complications. The early death rate in TAM ranges between 11 and 23% (118–120). Typical clinical signs are leukocytosis, anemia, thrombocytopenia, and hepatosplenomegaly as an indicator of liver infiltration and fibrosis (25, 116, 119, 120). In severe cases, the progressive infiltration can cause liver failure accompanied by coagulopathy (121). TAM can also lead to hydrops fetalis and cause the miscarriage of DS fetuses (122–127).

In the majority of TAM patients, the course of disease is self-limiting. However, intervention is needed for individuals with severe TAM-related clinical symptoms, which carry a high risk of causing early death. Since the hyperproliferative TAM blasts are very susceptible to cytarabine, various studies investigated the use of cytarabine in high risk TAM patients (25, 116, 128). Although the application of cytarabine might increase survival rates in TAM patients with critical disease, the dosing scheme and indications for chemotherapy differ between study groups (25, 116, 128, 129).

With about 13–33%, a high portion of TAM patients progress to ML–DS, usually before they reach the age of four years (2, 116, 118, 130). Unfortunately, measurable residual disease (MRD)-monitored low-dose cytarabine treatment of TAM patients was not able to reduce this high progression rate (25, 129).

## From TAM to ML–DS: Clonal Evolution in DS Leukemogenesis

Once progressed from TAM to ML–DS, the disease course is no longer self-limiting, and all patients need intensive chemotherapy to achieve long-term survival (5).

Evolution from TAM to ML–DS seems to depend on the acquisition of additional mutations in persistent *GATA1* mutant cells. Somatic mutations in ML–DS patients most frequently affect cohesin complex genes, JAK family kinases, and epigenetic regulators, but mutations frequently observed in AML, such as *FLT3* or *TP53* mutations, can also be found (**Table 1**) (26–28). While TAM samples harbor on average 0.4 mutations in addition to the *GATA1s* mutation, ML–DS samples had 1.6 detectable variants per sample (26). Although at a low frequency, some TAM patients were shown to harbor somatic variants in addition to the *GATA1s* mutation. However, “third hit” TAM mutations were not necessarily associated with progression to ML–DS (27).

**Table 1:** Summary of somatic mutations identified in ML–DS samples in addition to the mandatory *GATA1s* mutation.

	Genes mutated	Frequency of mutation in different studies (%)	References
**cohesin complex**	*CTCF*	16/141 (11.3); 10/49 (20.4)	(26, 28)
	*NIPBL*	5/141 (3.5); 3/49 (6.1)	(26, 28)
	*RAD21*	16/141 (11.3); 11/49 (22.4)	(26, 28)
	*SMC1A*	9/141 (6.4); 2/49 (4.1)	(26, 28)
	*SMC3*	1/141 (0.7); 1/7 (14.3); 1/49 (2.0)	(26–28)
	*STAG2*	19/141 (13.5); 9/49 (18.4)	(26, 28)
**epigenetic regulators**	*ASXL1*	1/49 (2.0)	(28)
	*BCOR*	2/141 (1.4); 2/49 (4.1)	(26, 28)
	*DNMT3A*	1/49 (2.0)	(28)
	*EED*	1/141 (0.7)	(26)
	*EP300*	1/141 (0.7)	(26)
	*EZH2*	10/141 (7.1); 1/7 (14.3); 16/49 (32.7)	(26–28)
	*KANSL1*	17/141 (12.1); 3/49 (6.1)	(26, 28)
	*KDM6A*	1/141 (0.7)	(26)
	*KMT2C*	1/141 (0.7)	(26)
	*NAT6*	1/141 (0.7)	(26)
	*SUZ12*	9/141 (6.4); 1/49 (2.0)	(26, 28)
	*TET2*	2/141 (1.4)	(26)
**tyrosine kinases**	*GNB1*	1/141 (0.7)	(26)
	*JAK1*	6/141 (4.3); 1/7 (14.3); 2/49 (4.1)	(26–28)
	*JAK2*	14/141 (9.9); 4/49 (8.2); 1/7 (14.3)	(26, 28, 131)
	*JAK3*	19/141 (13.5); 6/49 (12.2); 2/13 (15.4); 1/3 (33.3); 1/7 (14.3); 1/14 (7.1)	(26, 28, 131–134)
	*KIT*	2/141 (1.4)	(26)
	*MPL*	10/141 (7.1); 3/49 (6.1)	(26, 28)
	*PTEN*	1/141 (0.7)	(26)
	*PTPRD*	1/141 (0.7)	(26)
	*SH2B3*	4/141 (2.8); 4/49 (8.2)	(26, 28)
**RAS**	*KRAS*	7/141 (5.0); 4/49 (8.2)	(26, 28)
	*NF1*	4/141 (2.8)	(26)
	*NRAS*	6/141 (4.3); 4/49 (8.2)	(26, 28)
	*PTPN11*	1/49 (2.0)	(28)
**transcription factors**	*CREBBP*	1/141 (0.7)	(26)
	*FLT3*	1/7 (14.3); 2/7 (28.6)	(27, 131)
	*MYC*	1/141 (0.7)	(26)
	*RUNX1*	3/141 (2.1)	(26)
	*TP53*	5/141 (3.5); 3/49 (6.1); 2/13 (15.4)	(26, 28, 132)
	*WT1*	1/141 (0.7); 2/49 (4.1)	(26, 28)
**others**	*CSF2RB*	7/141 (5.0)	(26)
	*DCAF7*	1/141 (0.7); 2/49 (4.1)	(26, 28)
	*DLEC1*	1/7 (14.3)	(27)
	*DHX29*	1/7 (14.3)	(27)
	*PI3KC2A*	1/7 (14.3)	(27)
	*POLE*	1/7 (14.3)	(27)
	*SF3B1*	3/141 (2.1)	(26)
	*SRSF2*	12/141 (8.5); 1/49 (2.0)	(26, 28)
**chromosomal aberrations**	del(5q)	3/141 (2.1); 1/7 (14.3)	(26, 27)
	tetrasomy 14	1/7 (14.3)	(27)
	tetrasomy 21	1/7 (14.3)	(27)
	i(7q)	1/7 (14.3)	(27)
	submicroscopic del(8q)	1/7 (14.3)	(27)
	submicroscopic del(6q)	1/7 (14.3)	(27)
	trisomy 8	1/7 (14.3)	(131)
	inv (9)(p11;q12)	1/7 (14.3)	(131)
	complex karyotype	1/7 (14.3)	(131)

### Mutations in Cohesin Complex Genes

The cohesin complex is essential for the controlled course of mitosis, as it holds the replicated chromosomes together during metaphase. Still, in recent years, the role of the cohesin complex in transcriptional regulation has been recognized, as it organizes higher order chromatin structure. It was shown that the cohesin complex brings together enhancer and promoter regions by forming DNA loops (135). In this process, CTCF is known to cooperate with the cohesin complex to form DNA loops within topologically associated domains (130, 135).

Mutations in the four main components of the cohesin complex—SMC1, SMC2, RAD21, STAG2—are frequently found in ML–DS patients, but also in other myeloid neoplasms and solid cancers (26, 28). Cohesin complex mutations are mutually exclusive and in the majority of cases are loss of function mutations (136, 137). Additionally, recurrent *CTCF* mutations were identified, which are unique to the molecular landscape of ML–DS (28). In a murine GATA1s model using CRISPR-Cas9 to recreate the clonal evolution from TAM to ML–DS, cohesin complex and *Ctcf* loss-of-function mutations were significantly underrepresented compared to human ML–DS samples (26). Since this model lacked the presence of T21, these data might underline the importance of a T21 genetic background for the oncogenic effect of cohesin complex mutations. Moreover, species-specific functions of the cohesin complex during hematopoiesis cannot be excluded—an alternative explanation that requires further investigations. Previously, cohesin complex mutations were demonstrated to block differentiation in human HSPCs while increasing their self-renewal capacity, in line with data from a murine model (138, 139). Consistently, loss of *rad21* in zebrafish causes impaired hematopoiesis during embryonic development by preventing the expression of *runx1* (140). Deletion of *Smc3* resulted in severe pancytopenia and 100% mortality in mice (141). Of note, haploinsufficiency of *Smc3* led to a proliferative advantage over *Smc3* wild-type bone marrow cells and cooperated with *Flt3*-ITD in AML progression (141).

In addition to transcriptional regulation through the looping of DNA, changes in chromatin accessibility were observed in cohesin mutant and knock-down models (138, 139, 141). In contrast to a global reduction of chromatin accessibility, ERG, RUNX1, and GATA2 motifs displayed increased accessibility in cohesin mutant cells, suggesting that these transcription factors—which are also implicated in DS leukemogenesis—largely contribute to an enhanced transcriptional stemness program observed in cohesin mutant HSPCs (138).

### Mutations in JAK-STAT-Signaling Pathways

Activating mutations in the tyrosine kinases JAK1, JAK2, and JAK3 were previously identified in AMKL in individuals with or without DS (26, 131–133, 142, 143). However, some variants seem to be exclusive to ML-DS (134). Interestingly, activating mutations were only identified in ML-DS, while the significance of JAK mutations in TAM samples was unknown or the variant caused loss of function, suggesting that aberrant activation of JAK-STAT signaling is essential for leukemic transformation in ML–DS (26).

Recently, a new hotspot mutation in *CSF2RB* was identified in ML-DS samples (26). *CSF2RB* encodes the common β chain of various cytokine receptors, which activate downstream JAK-STAT and other pathways. The *CSF2RB^A455D^* variant is predicted to lead to a constitutively active cytokine receptor due to aberrant dimerization of the transmembrane domains of two *β* chains or an *α* and a *β* chain—a hypothesis which is supported by the cytokine independent growth of TF1 cells harboring the *CSF2RB^A455D^* mutant (26). When the *CSF2RB^A455D^* mutant was expressed in HSPCs, a differentiation block in terminal megakaryopoiesis along with an expansion of immature erythroid cells was observed (26). These changes were reversed upon treatment with the JAK inhibitor Ruxolitinib, suggesting that the oncogenic potential of the *CSF2RB^A455D^* variant manifests in aberrant JAK-STAT-signaling. Further work showed that *CSF2RB^A455D^* activates TPOR, to drive pathogenic TPOR signaling in ML–DS (144).

### Mutations in Epigenetic Modifiers and Altered DNA Methylation

ML–DS samples were shown to harbor a genome-wide pattern of hypomethylation discriminating them from non-DS AMKL, which in comparison displayed hypermethylation at the analyzed differentially methylated regions (145). Thus, this global hypomethylation seems to be driven by T21. Upon acquisition of *GATA1s* mutations certain genetic regions gain aberrant hypermethylation compared to T21-*GATA1^wild-type^* fetal liver cells and pathway analyses revealed that gene networks involved in cell cycle, cell signaling and proliferation were especially affected by this local hypermethylation implying functional relevance of these differentially methylated regions (145).

The importance of epigenetic changes for leukemic transformation in individuals with TAM is also underlined by the frequent identification of mutations in epigenetic modifiers, such as *EZH2*, *KANSL1*, and *SUZ12* (26, 28). However, the spectrum of mutated epigenetic regulators in ML–DS largely differs from the genes that are frequently mutated in adult AML, such as *DNMT3A*, *IDH1*, *IDH2*, and *TET2*.

### Role of Non-Coding RNAs in the Development of ML–DS

MicroRNAs play pivotal roles as post-transcriptional regulators in leukemogenesis. We already discussed the impact of *miR-125b-2*, which is located on chromosome 21 and promotes megakaryocytic expansion upon increased gene dosage in T21 individuals (73).

Another microRNA implicated in DS leukemogenesis is *miR-486*, which is encoded within its host gene *ANK1* on chromosome 8. *ANK1* is a known target gene of GATA1 and *miR-486* levels were directly correlated with *GATA1s* expression in ML-DS samples (146). Overexpression of *miR-486* alone failed to transform fetal liver cells, but increased self-renewal capacity when expressed together with *GATA1s* (146). The oncogenic potential of *miR-486* might be exerted through activation of the PI3K-AKT pathway.

The importance of non-coding RNAs in DS leukemogenesis was also recently underlined by a large sequencing study analyzing samples of normal hematopoietic cells and different AML subgroups, such as ML–DS (147). It was shown that ML-DS samples harbor a non-coding RNA signature with similarities to healthy HSCs, characterized by the down-regulation of non-coding RNAs associated with differentiation (147). The lncRNAs *MONC* and *MIR100HG* are the host genes of the *miR-99a~125b-2* tricistron and its homolog on chromosome 11, respectively and were also implicated in AMKL and ML–DS pathogenesis, as knock-down of both lncRNAs resulted in reduced proliferation in corresponding leukemic cell lines (148). Interestingly, overexpression of a spliced form of *MONC* in HSPCs caused an erythroid lineage bias and expansion of immature erythroid cells independent from the *miR-99a~125b-2* tricistron (148).

### Chromosomal Aberrations as Drivers for ML-DS Progression

In addition to the discussed point and indel mutations, the acquisition of structural chromosomal aberrations was also observed in ML–DS samples. These range from small submicroscopic deletions to tetrasomy of whole chromosomes (27). For instance, partial deletions of two regions of chromosome 5, which are usually not affected in other hematopoietic malignancies such as 5q-myelodysplastic syndrome, were found and resulted in deletion of the tumor suppressor APC (27).

As opposed to ML–DS samples, copy number alterations are hardly observed at the TAM stage, pointing to the transformative character of these genomic changes (27, 149). However, another study involving serial transplantations of primary TAM samples suggested that structural chromosomal changes may be already present in very small TAM subclones and expand due to positive selection upon progression to ML–DS (149).

## Concluding Remarks

T21 predisposes individuals to the development of ML–DS, with pre-leukemic TAM already originating *in utero*. ML–DS displays a step-wise model of leukemogenesis offering the unique opportunity to investigate clonal evolution in myeloid leukemias.

As discussed in this review, the first step in DS leukemogenesis is the disruption of hematopoietic transcription factor networks resulting from the increased gene dosages of some members of these circuits due to T21, consequently leading to megakaryocytic expansion (**Figure 2**). The acquisition of truncating *GATA1* mutations in this susceptible, highly proliferating cell population during fetal liver hematopoiesis marks the second step in DS leukemogenesis (**Figure 2**). Upon exclusive GATA1s expression, dysplastic megakaryocytic cells undergo uncontrolled expansion, accompanied by disrupted erythroid differentiation. Again, this aberrant proliferation facilitates the acquisition and positive selection of clones with additional somatic mutations, ultimately paving the way to progression to ML–DS.

Strikingly, recent studies in pediatric non-DS-AMKL also pointed towards developmental stage-specific effects of fusion genes essential to this entity (150) and suggested a fetal origin of the disease (150–152) similar to ML–DS.

In DS leukemogenesis the developmental stage-specific effects of T21 and *GATA1s* mutations and the impact of the fetal liver microenvironment, both contribute to the self-limiting nature of TAM in the majority of patients. However, further work needs to be done to better characterize the molecular synergy between T21 and GATA1s in driving ML-DS development, and to unravel the transformative features of additional somatic mutations, as not all TAM individuals with “third hit” mutations progress to ML–DS. The insights gained from such studies will shed light onto the mechanisms of genetic predisposition to cancer development, which can also be extrapolated to other entities, making ML–DS a valuable model of leukemogenesis.

## Author Contributions

JG drafted the manuscript. DH and JHK revised the content and approved the manuscript for publication. All authors contributed to the article and approved the submitted version.

## Funding

This work was supported by funding to JHK from the European Research Council (ERC) under the European Union’s Horizon 2020 research and innovation program (grant agreement #714226). JHK is a recipient of the St. Baldrick’s Robert J. Arceci Innovation Award. DH is supported by the German Cancer Aid (#111743).

## Conflict of Interest

The authors declare that the research was conducted in the absence of any commercial or financial relationships that could be construed as a potential conflict of interest.

## References

[1] Satgé D Seidel MG. The Pattern of Malignancies in Down Syndrome and Its Potential Context With the Immune System. Front Immunol (2018) 9:3058. 10.3389/fimmu.2018.03058 30631328 PMC6315194

[2] Hasle H. Pattern of malignant disorders in individuals with Down’s syndrome. Lancet Oncol (2001) 2:429–36. 10.1016/S1470-2045(00)00435-6 11905737

[3] Hasle H Clemmensen IH Mikkelsen M. Risks of leukaemia and solid tumours in individuals with Down’s syndrome. Lancet (2000) 355:165–9. 10.1016/S0140-6736(99)05264-2 10675114

[4] Hasle H Friedman JM Olsen JH Rasmussen SA. Low risk of solid tumors in persons with Down syndrome. Genet Med (2016) 18:1151–7. 10.1038/gim.2016.23 27031084

[5] Uffmann M Rasche M Zimmermann M von Neuhoff C Creutzig U Dworzak M. Therapy reduction in patients with Down syndrome and myeloid leukemia: The international ML-DS 2006 trial. Blood (2017) 129:3314–21. 10.1182/blood-2017-01-765057 28400376

[6] Byrska-Bishop M Vandorn D Campbell AE Betensky M Arca PR Yao Y. Pluripotent stem cells reveal erythroid-specific activities of the GATA1 N-terminus. J Clin Invest (2015) 125:993–1005. 10.1172/JCI75714 25621499 PMC4362246

[7] Chou ST Opalinska JB Yao Y Fernandes MA Kalota A Brooks JS. Trisomy 21 enhances human fetal erythro-megakaryocytic development. Blood (2008) 112:4503–6. 10.1182/blood-2008-05-157859 PMC2597125 18812473

[8] Maclean GA Menne TF Guo G Sanchez DJ Park I-H Daley GQ. Altered hematopoiesis in trisomy 21 as revealed through in vitro differentiation of isogenic human pluripotent cells. Proc Natl Acad Sci U S A (2012) 109:17567–72. 10.1073/pnas.1215468109 PMC3491455 23045682

[9] Roy A Cowan G Mead AJ Filippi S Bohn G Chaidos A. Perturbation of fetal liver hematopoietic stem and progenitor cell development by trisomy 21. Proc Natl Acad Sci U S A (2012) 109:17579–84. 10.1073/pnas.1211405109 PMC3491522 23045701

[10] Tunstall-Pedoe O Roy A Karadimitris A La de FJ Fisk NM Bennett P. Abnormalities in the myeloid progenitor compartment in Down syndrome fetal liver precede acquisition of GATA1 mutations. Blood (2008) 112:4507–11. 10.1182/blood-2008-04-152967 18689547

[11] Ahmed M Sternberg A Hall G Thomas A Smith O O’Marcaigh A. Natural history of GATA1 mutations in Down syndrome. Blood (2004) 103:2480–9. 10.1182/blood-2003-10-3383 14656875

[12] Alford KA Reinhardt K Garnett C Norton A Böhmer K von NC. Analysis of GATA1 mutations in Down syndrome transient myeloproliferative disorder and myeloid leukemia. Blood (2011) 118:2222–38. 10.1182/blood-2011-03-342774 21715302

[13] Carpenter E Valverde-Garduno V Sternberg A Mitchell C Roberts I Vyas P. GATA1 mutation and trisomy 21 are required only in haematopoietic cells for development of transient myeloproliferative disorder. Br J Haematol (2005) 128:548–51. 10.1111/j.1365-2141.2004.05342.x 15686466

[14] Greene ME Mundschau G Wechsler J McDevitt M Gamis A Karp J. Mutations in GATA1 in both transient myeloproliferative disorder and acute megakaryoblastic leukemia of Down syndrome. Blood Cells Mol Dis (2003) 31:351–6. 10.1016/j.bcmd.2003.08.001 14636651

[15] Groet J McElwaine S Spinelli M Rinaldi A Burtscher I Mulligan C. Acquired mutations in GATA1 in neonates with Down’s syndrome with transient myeloid disorder. Lancet (2003) 361:1617–20. 10.1016/S0140-6736(03)13266-7 12747884

[16] Hitzler JK Cheung J Li Y Scherer SW Zipursky A. GATA1 mutations in transient leukemia and acute megakaryoblastic leukemia of Down syndrome. Blood (2003) 101:4301–4. 10.1182/blood-2003-01-0013 12586620

[17] Mundschau G Gurbuxani S Gamis AS Greene ME Arceci RJ Crispino JD. Mutagenesis of GATA1 is an initiating event in Down syndrome leukemogenesis. Blood (2003) 101:4298–300. 10.1182/blood-2002-12-3904 12560215

[18] Rainis L Bercovich D Strehl S Teigler-Schlegel A Stark B Trka J. Mutations in exon 2 of GATA1 are early events in megakaryocytic malignancies associated with trisomy 21. Blood (2003) 102:981–6. 10.1182/blood-2002-11-3599 12649131

[19] Wechsler J Greene M McDevitt MA Anastasi J Karp JE Le Beau MM. Acquired mutations in GATA1 in the megakaryoblastic leukemia of Down syndrome. Nat Genet (2002) 32:148–52. 10.1038/ng955 12172547

[20] Xu G Nagano M Kanezaki R Toki T Hayashi Y Taketani T. Frequent mutations in the GATA-1 gene in the transient myeloproliferative disorder of Down syndrome. Blood (2003) 102:2960–8. 10.1182/blood-2003-02-0390 12816863

[21] Gialesaki S Mahnken AK Schmid L Labuhn M Bhayadia R Heckl D. GATA1s exerts developmental stage-specific effects in human hematopoiesis. Haematologica (2018) 103:e336–40. 10.3324/haematol.2018.191338 PMC6068023 29567780

[22] Kazuki Y Yakura Y Abe S Osaki M Kajitani N Kazuki K. Down syndrome-associated haematopoiesis abnormalities created by chromosome transfer and genome editing technologies. Sci Rep (2014) 4:6136. 10.1038/srep06136 25159877 PMC4145315

[23] Banno K Omori S Hirata K Nawa N Nakagawa N Nishimura K. Systematic Cellular Disease Models Reveal Synergistic Interaction of Trisomy 21 and GATA1 Mutations in Hematopoietic Abnormalities. Cell Rep (2016) 15:1228–41. 10.1016/j.celrep.2016.04.031 27134169

[24] Pine SR Guo Q Yin C Jayabose S Druschel CM Sandoval C. Incidence and clinical implications of GATA1 mutations in newborns with Down syndrome. Blood (2007) 110:2128–31. 10.1182/blood-2007-01-069542 17576817

[25] Klusmann JH Creutzig U Zimmermann M Dworzak M Jorch N Langebrake C. Treatment and prognostic impact of transient leukemia in neonates with Down syndrome. Blood (2008) 111:2991–8. 10.1182/blood-2007-10-118810 PMC2265448 18182574

[26] Labuhn M Perkins K Matzk S Varghese L Garnett C Papaemmanuil E. Mechanisms of Progression of Myeloid Preleukemia to Transformed Myeloid Leukemia in Children with Down Syndrome. Cancer Cell (2019) 36:123–38.e10. 10.1016/j.ccell.2019.06.007 31303423 PMC6863161

[27] Nikolaev SI Santoni F Vannier A Falconnet E Giarin E Basso G. Exome sequencing identifies putative drivers of progression of transient myeloproliferative disorder to AMKL in infants with Down syndrome. Blood (2013) 122:554–61. 10.1182/blood-2013-03-491936 23733339

[28] Yoshida K Toki T Okuno Y Kanezaki R Shiraishi Y Sato-Otsubo A. The landscape of somatic mutations in Down syndrome-related myeloid disorders. Nat Genet (2013) 45:1293–9. 10.1038/ng.2759 24056718

[29] Chou ST Byrska-Bishop M Tober JM Yao Y Vandorn D Opalinska JB. Trisomy 21-associated defects in human primitive hematopoiesis revealed through induced pluripotent stem cells. Proc Natl Acad Sci U S A (2012) 109:17573–8. 10.1073/pnas.1211175109 PMC3491490 23045704

[30] Alford KA Slender A Vanes L Li Z Fisher EM Nizetic D. Perturbed hematopoiesis in the Tc1 mouse model of Down syndrome. Blood (2010) 115:2928–37. 10.1182/blood-2009-06-227629 PMC2854435 20154221

[31] Carmichael CL Majewski IJ Alexander WS Metcalf D Hilton DJ Hewitt CA. Hematopoietic defects in the Ts1Cje mouse model of Down syndrome. Blood (2009) 113:1929–37. 10.1182/blood-2008-06-161422 19109561

[32] Malinge S Bliss-Moreau M Kirsammer G Diebold L Chlon T Gurbuxani S. Increased dosage of the chromosome 21 ortholog Dyrk1a promotes megakaryoblastic leukemia in a murine model of Down syndrome. J Clin Invest (2012) 122:948–62. 10.1172/JCI60455 PMC3287382 22354171

[33] Kirsammer G Jilani S Liu H Davis E Gurbuxani S Le Beau MM. Highly penetrant myeloproliferative disease in the Ts65Dn mouse model of Down syndrome. Blood (2008) 111:767–75. 10.1182/blood-2007-04-085670 PMC2200841 17901249

[34] Rahmani Z Blouin JL Creau-Goldberg N Watkins PC Mattei JF Poissonnier M. Critical role of the D21S55 region on chromosome 21 in the pathogenesis of Down syndrome. Proc Natl Acad Sci U S A (1989) 86:5958–62. 10.1073/pnas.86.15.5958 PMC297750 2527368

[35] Delabar JM Theophile D Rahmani Z Chettouh Z Blouin JL Prieur M. Molecular mapping of twenty-four features of Down syndrome on chromosome 21. Eur J Hum Genet (1993) 1:114–24. 10.1159/000472398 8055322

[36] McCormick MK Schinzel A Petersen MB Stetten G Driscoll DJ Cantu ES. Molecular genetic approach to the characterization of the “Down syndrome region” of chromosome 21. Genomics (1989) 5:325–31. 10.1016/0888-7543(89)90065-7 2529205

[37] Korenberg JR Kawashima H Pulst SM Ikeuchi T Ogasawara N Yamamoto K. Molecular definition of a region of chromosome 21 that causes features of the Down syndrome phenotype. Am J Hum Genet (1990) 47:236–46. PMC1683719 2143053

[38] Sinet PM Théophile D Rahmani Z Chettouh Z Blouin JL Prieur M. Mapping of the down syndrome phenotype on chromosome 21 at the molecular level. Biomed Pharmacother (1994) 48:247–52. 10.1016/0753-3322(94)90140-6 7999986

[39] Korenberg JR Chen XN Schipper R Sun Z Gonsky R Gerwehr S. Down syndrome phenotypes: The consequences of chromosomal imbalance. Proc Natl Acad Sci U S A (1994) 91:4997–5001. 10.1073/pnas.91.11.4997 8197171 PMC43917

[40] Lyle R Béna F Gagos S Gehrig C Lopez G Schinzel A. Genotype-phenotype correlations in Down syndrome identified by array CGH in 30 cases of partial trisomy and partial monosomy chromosome 21. Eur J Hum Genet (2009) 17:454–66. 10.1038/ejhg.2008.214 PMC2986205 19002211

[41] Barlow GM Chen XN Shi ZY Lyons GE Kurnit DM Celle L. Down syndrome congenital heart disease: A narrowed region and a candidate gene. Genet Med (2001) 3:91–101. 10.1097/00125817-200103000-00002 11280955

[42] Korbel JO Tirosh-Wagner T Urban AE Chen X-N Kasowski M Dai L. The genetic architecture of Down syndrome phenotypes revealed by high-resolution analysis of human segmental trisomies. Proc Natl Acad Sci U S A (2009) 106:12031–6. 10.1073/pnas.0813248106 PMC2709665 19597142

[43] Tracey WD Speck NA. Potential roles for RUNX1 and its orthologs in determining hematopoietic cell fate. Semin Cell Dev Biol (2000) 11:337–42. 10.1006/scdb.2000.0186 11105897

[44] Okuda T van Deursen J Hiebert SW Grosveld G Downing JR. AML1, the Target of Multiple Chromosomal Translocations in Human Leukemia, Is Essential for Normal Fetal Liver Hematopoiesis. Cell (1996) 84:321–30. 10.1016/s0092-8674(00)80986-1 8565077

[45] Wang Q Stacy T Binder M Marin-Padilla M Sharpe AH Speck NA. Disruption of the Cbfa2 gene causes necrosis and hemorrhaging in the central nervous system and blocks definitive hematopoiesis. Proc Natl Acad Sci U S A (1996) 93:3444–9. 10.1073/pnas.93.8.3444 PMC39628 8622955

[46] Miyoshi H Ohira M Shimizu K Mitani K Hirai H Imai T. Alternative splicing and genomic structure of the AML1 gene involved in acute myeloid leukemia. Nucleic Acids Res (1995) 23:2762–9. 10.1093/nar/23.14.2762 PMC307102 7651838

[47] Liu X Zhang Q Zhang D-E Zhou C Xing H Tian Z. Overexpression of an isoform of AML1 in acute leukemia and its potential role in leukemogenesis. Leukemia (2009) 23:739–45. 10.1038/leu.2008.350 19151769

[48] Peterson LF Zhang D-E. The 8;21 translocation in leukemogenesis. Oncogene (2004) 23:4255–62. 10.1038/sj.onc.1207727 15156181

[49] Elagib KE Racke FK Mogass M Khetawat R Delehanty LL Goldfarb AN. RUNX1 and GATA-1 coexpression and cooperation in megakaryocytic differentiation. Blood (2003) 101:4333–41. 10.1182/blood-2002-09-2708 12576332

[50] Rainis L Toki T Pimanda JE Rosenthal E Machol K Strehl S. The proto-oncogene ERG in megakaryoblastic leukemias. Cancer Res (2005) 65:7596–602. 10.1158/0008-5472.CAN-05-0147 16140924

[51] Samir T Bee T Hilton A Knezevic K Scott J Willson TA. ERG dependence distinguishes developmental control of hematopoietic stem cell maintenance from hematopoietic specification. Genes Dev (2011) 25:251–62. 10.1101/gad.2009211 PMC3034900 21245161

[52] Loughran SJ Kruse EA Hacking DF de Graaf CA Hyland CD Willson TA. The transcription factor Erg is essential for definitive hematopoiesis and the function of adult hematopoietic stem cells. Nat Immunol (2008) 9:810–9. 10.1038/ni.1617 18500345

[53] Stankiewicz MJ Crispino JD. ETS2 and ERG promote megakaryopoiesis and synergize with alterations in GATA-1 to immortalize hematopoietic progenitor cells. Blood (2009) 113:3337–47. 10.1182/blood-2008-08-174813 PMC2665899 19168790

[54] Xie Y Koch ML Zhang X Hamblen MJ Godinho FJ Fujiwara Y. Reduced Erg Dosage Impairs Survival of Hematopoietic Stem and Progenitor Cells. Stem Cells (2017) 35:1773–85. 10.1002/stem.2627 PMC5532742 28436588

[55] Baldus CD Liyanarachchi S Mrózek K Auer H Tanner SM Guimond M. Acute myeloid leukemia with complex karyotypes and abnormal chromosome 21: Amplification discloses overexpression of APP, ETS2, and ERG genes. Proc Natl Acad Sci U S A (2004) 101:3915–20. 10.1073/pnas.0400272101 PMC374344 15007164

[56] Ge Y LaFiura KM Dombkowski AA Chen Q Payton SG Buck SA. The role of the proto-oncogene ETS2 in acute megakaryocytic leukemia biology and therapy. Leukemia (2008) 22:521–9. 10.1038/sj.leu.2405066 PMC3809919 18094719

[57] Eisbacher M Holmes ML Newton A Hogg PJ Khachigian LM Crossley M. Protein-protein interaction between Fli-1 and GATA-1 mediates synergistic expression of megakaryocyte-specific genes through cooperative DNA binding. Mol Cell Biol (2003) 23:3427–41. 10.1128/mcb.23.10.3427-3441.2003 PMC154245 12724402

[58] Gosiengfiao Y Horvat R Thompson A. Transcription factors GATA-1 and Fli-1 regulate human HOXA10 expression in megakaryocytic cells. DNA Cell Biol (2007) 26:577–87. 10.1089/dna.2007.0575 17688409

[59] Birger Y Goldberg L Chlon TM Goldenson B Muler I Schiby G. Perturbation of fetal hematopoiesis in a mouse model of Down syndrome’s transient myeloproliferative disorder. Blood (2013) 122:988–98. 10.1182/blood-2012-10-460998 PMC3739041 23719302

[60] Crabtree GR Olson EN. NFAT Signaling. Cell (2002) 109:S67–79. 10.1016/S0092-8674(02)00699-2 11983154

[61] Graef IA Chen F Crabtree GR. NFAT signaling in vertebrate development. Curr Opin Genet Dev (2001) 11:505–12. 10.1016/S0959-437X(00)00225-2 11532391

[62] Nguyen T Di Giovanni S. NFAT signaling in neural development and axon growth. Int J Dev Neurosci (2008) 26:141–5. 10.1016/j.ijdevneu.2007.10.004 PMC2267928 18093786

[63] Macian F. NFAT proteins: Key regulators of T-cell development and function. Nat Rev Immunol (2005) 5:472–84. 10.1038/nri1632 15928679

[64] Arron JR Winslow MM Polleri A Chang CP Wu H Gao X. NFAT dysregulation by increased dosage of DSCR1 and DYRK1A on chromosome 21. Nature (2006) 441:595–600. 10.1038/nature04678 16554754

[65] Flanagan WM Corthésy B Bram RJ Crabtree GR. Nuclear association of a T-cell transcription factor blocked by FK-506 and cyclosporin A. Nature (1991) 352:803–7. 10.1038/352803a0 1715516

[66] Beals CR Sheridan CM Turck CW Gardner P Crabtree GR. Nuclear export of NF-ATc enhanced by glycogen synthase kinase-3. Science (1997) 275:1930–4. 10.1126/science.275.5308.1930 9072970

[67] Graef IA Mermelstein PG Stankunas K Neilson JR Deisseroth K Tsien RW. L-type calcium channels and GSK-3 regulate the activity of NF-ATc4 in hippocampal neurons. Nature (1999) 401:703–8. 10.1038/44378 10537109

[68] Northrop JP Ho SN Chen L Thomas DJ Timmerman La Nolan GP. NF-AT components define a family of transcription factors targeted in T-cell activation. Nature (1994) 369:497–502. 10.1038/369497a0 8202141

[69] Krude T. Chromatin assembly factor 1 (CAF-1) colocalizes with replication foci in HeLa cell nuclei. Exp Cell Res (1995) 220:304–11. 10.1006/excr.1995.1320 7556438

[70] Marheineke K Krude T. Nucleosome assembly activity and intracellular localization of human CAF-1 changes during the cell division cycle. J Biol Chem (1998) 273:15279–86. 10.1074/jbc.273.24.15279 9614144

[71] Volk A Liang K Suraneni P Li X Zhao J Bulic M. A CHAF1B-Dependent Molecular Switch in Hematopoiesis and Leukemia Pathogenesis. Cancer Cell (2018) 34:707–23.e7. 10.1016/j.ccell.2018.10.004 30423293 PMC6235627

[72] Carthew RW Sontheimer EJ. Origins and Mechanisms of miRNAs and siRNAs. Cell (2009) 136:642–55. 10.1016/j.cell.2009.01.035 PMC2675692 19239886

[73] Klusmann JH Li Z Böhmer K Maroz A Koch ML Emmrich S. miR-125b-2 is a potential oncomiR on human chromosome 21 in megakaryoblastic leukemia. Genes Dev (2010) 24:478–90. 10.1101/gad.1856210 PMC2827843 20194440

[74] Emmrich S Rasche M Schöning J Reimer C Keihani S Maroz A. miR-99a/100~125b tricistrons regulate hematopoietic stem and progenitor cell homeostasis by shifting the balance between TGFβ and Wnt signaling. Genes Dev (2014) 28:858–74. 10.1101/gad.233791.113 PMC4003278 24736844

[75] Kanezaki R Toki T Terui K Xu G Wang R Shimada A. Down syndrome and GATA1 mutations in transient abnormal myeloproliferative disorder: Mutation classes correlate with progression to myeloid leukemia. Blood (2010) 116:4631–8. 10.1182/blood-2010-05-282426 20729467

[76] Gruber TA Downing JR. The biology of pediatric acute megakaryoblastic leukemia. Blood (2015) 126:943–9. 10.1182/blood-2015-05-567859 PMC4551356 26186939

[77] Bresnick EH Katsumura KR Lee H-Y Johnson KD Perkins AS. Master regulatory GATA transcription factors: Mechanistic principles and emerging links to hematologic malignancies. Nucleic Acids Res (2012) 40:5819–31. 10.1093/nar/gks281 PMC3401466 22492510

[78] Crispino JD Horwitz MS. GATA factor mutations in hematologic disease. Blood (2017) 129:2103–10. 10.1182/blood-2016-09-687889 PMC5391620 28179280

[79] Evans T Reitman M Felsenfeld G. An erythrocyte-specific DNA-binding factor recognizes a regulatory sequence common to all chicken globin genes. Proc Natl Acad Sci U S A (1988) 85:5976–80. 10.1073/pnas.85.16.5976 PMC281888 3413070

[80] Martin DI Tsai SF Orkin SH. Increased gamma-globin expression in a nondeletion HPFH mediated by an erythroid-specific DNA-binding factor. Nature (1989) 338. 10.1038/338435a0 2467208

[81] Wall L deBoer E Grosveld F. The human beta-globin gene 3’ enhancer contains multiple binding sites for an erythroid-specific protein. Genes Dev (1988) 2:1089–100. 10.1101/gad.2.9.1089 2461328

[82] Tsang AP Visvader JE Turner CA Fujiwara Y Yu C Weiss MJ. FOG, a multitype zinc finger protein, acts as a cofactor for transcription factor GATA-1 in erythroid and megakaryocytic differentiation. Cell (1997) 90:109–19. 10.1016/s0092-8674(00)80318-9 9230307

[83] Orkin SH Zon LI. Hematopoiesis: an evolving paradigm for stem cell biology. Cell (2008) 132:631–44. 10.1016/j.cell.2008.01.025 PMC2628169 18295580

[84] Ohneda K Yamamoto M. Roles of hematopoietic transcription factors GATA-1 and GATA-2 in the development of red blood cell lineage. Acta Haematol (2002) 108:237–45. 10.1159/000065660 12432220

[85] Grass JA Jing H Kim S-I Martowicz ML Pal S Blobel GA. Distinct functions of dispersed GATA factor complexes at an endogenous gene locus. Mol Cell Biol (2006) 26:7056–67. 10.1128/MCB.01033-06 PMC1592882 16980610

[86] Takai J Moriguchi T Suzuki M Yu L Ohneda K Yamamoto M. The Gata1 5’ region harbors distinct cis-regulatory modules that direct gene activation in erythroid cells and gene inactivation in HSCs. Blood (2013) 122:3450–60. 10.1182/blood-2013-01-476911 24021675

[87] Ling T Birger Y Stankiewicz MJ Ben-Haim N Kalisky T Rein A. Chromatin occupancy and epigenetic analysis reveal new insights into the function of the GATA1 N terminus in erythropoiesis. Blood (2019) 134:1619–31. 10.1182/blood.2019001234 PMC6871310 31409672

[88] Fujiwara Y Browne CP Cunniff K Goff SC Orkin SH. Arrested development of embryonic red cell precursors in mouse embryos lacking transcription factor GATA-1. Proc Natl Acad Sci U S A (1996) 93:12355–8. 10.1073/pnas.93.22.12355 PMC37995 8901585

[89] Gregory T Yu C Ma A Orkin SH Blobel GA Weiss MJ. GATA-1 and erythropoietin cooperate to promote erythroid cell survival by regulating bcl-xL expression. Blood (1999) 94:87–96. 10381501

[90] Gutiérrez L Tsukamoto S Suzuki M Yamamoto-Mukai H Yamamoto M Philipsen S. Ablation of Gata1 in adult mice results in aplastic crisis, revealing its essential role in steady-state and stress erythropoiesis. Blood (2008) 111:4375–85. 10.1182/blood-2007-09-115121 18258797

[91] Weiss MJ Orkin SH. Transcription factor GATA-1 permits survival and maturation of erythroid precursors by preventing apoptosis. Proc Natl Acad Sci U S A (1995) 92:9623–7. 10.1073/pnas.92.21.9623 PMC40854 7568185

[92] McDevitt MA Shivdasani RA Fujiwara Y Yang H Orkin SH. A “knockdown” mutation created by cis-element gene targeting reveals the dependence of erythroid cell maturation on the level of transcription factor GATA-1. Proc Natl Acad Sci U S A (1997) 94:6781–5. 10.1073/pnas.94.13.6781 PMC21235 9192642

[93] Pan X Ohneda O Ohneda K Lindeboom F Iwata F Shimizu R. Graded levels of GATA-1 expression modulate survival, proliferation, and differentiation of erythroid progenitors. J Biol Chem (2005) 280:22385–94. 10.1074/jbc.M500081200 15817467

[94] Vyas P Ault K Jackson CW Orkin SH Shivdasani RA. Consequences of GATA-1 deficiency in megakaryocytes and platelets. Blood (1999) 93:2867–75. 10216081

[95] Shivdasani RA Fujiwara Y McDevitt MA Orkin SH. A lineage-selective knockout establishes the critical role of transcription factor GATA-1 in megakaryocyte growth and platelet development. EMBO J (1997) 16:3965–73. 10.1093/emboj/16.13.3965 PMC1170020 9233806

[96] Nichols KE Crispino JD Poncz M White JG Orkin SH Maris JM. Familial dyserythropoietic anaemia and thrombocytopenia due to an inherited mutation in GATA1. Nat Genet (2000) 24:266–70. 10.1038/73480 PMC10576470 10700180

[97] Crispino JD Weiss MJ. Erythro-megakaryocytic transcription factors associated with hereditary anemia. Blood (2014) 123:3080–8. 10.1182/blood-2014-01-453167 PMC4023417 24652993

[98] Sankaran VG Ghazvinian R Do R Thiru P Vergilio J-A Beggs AH. Exome sequencing identifies GATA1 mutations resulting in Diamond-Blackfan anemia. J Clin Invest (2012) 122:2439–43. 10.1172/JCI63597 PMC3386831 22706301

[99] Parrella S Aspesi A Quarello P Garelli E Pavesi E Carando A. Loss of GATA-1 full length as a cause of Diamond-Blackfan anemia phenotype. Pediatr Blood Cancer (2014) 61:1319–21. 10.1002/pbc.24944 PMC4684094 24453067

[100] Hollanda LM Lima CS Cunha AF Albuquerque DM Vassallo J Ozelo MC. An inherited mutation leading to production of only the short isoform of GATA-1 is associated with impaired erythropoiesis. Nat Genet (2006) 38:807–12. 10.1038/ng1825 16783379

[101] Lukes J Danek P Alejo-Valle O Potuckova E Gahura O Heckl D. Chromosome 21 gain is dispensable for transient myeloproliferative disorder driven by a novel GATA1 mutation. Leukemia (2020) 34:2503–8. 10.1038/s41375-020-0769-1 32094462

[102] Bourquin J-P Subramanian A Langebrake C Reinhardt D Bernard O Ballerini P. Identification of distinct molecular phenotypes in acute megakaryoblastic leukemia by gene expression profiling. Proc Natl Acad Sci U S A (2006) 103:3339–44. 10.1073/pnas.0511150103 PMC1413912 16492768

[103] Li Z Godinho FJ Klusmann J-H Garriga-Canut M Yu C Orkin SH. Developmental stage-selective effect of somatically mutated leukemogenic transcription factor GATA1. Nat Genet (2005) 37:613–9. 10.1038/ng1566 15895080

[104] Klusmann JH Godinho FJ Heitmann K Maroz A Koch ML Reinhardt D. Developmental stage-specific interplay of GATA1 and IGF signaling in fetal megakaryopoiesis and leukemogenesis. Genes Dev (2010) 24:1659–72. 10.1101/gad.1903410 PMC2912563 20679399

[105] Kadri Z Shimizu R Ohneda O Maouche-Chretien L Gisselbrecht S Yamamoto M. Direct binding of pRb/E2F-2 to GATA-1 regulates maturation and terminal cell division during erythropoiesis. PloS Biol (2009) 7:e1000123. 10.1371/journal.pbio.1000123 19513100 PMC2684697

[106] Campbell AE Wilkinson-White L Mackay JP Matthews JM Blobel GA. Analysis of disease-causing GATA1 mutations in murine gene complementation systems. Blood (2013) 121:5218–27. 10.1182/blood-2013-03-488080 PMC3695365 23704091

[107] Muntean AG Crispino JD. Differential requirements for the activation domain and FOG-interaction surface of GATA-1 in megakaryocyte gene expression and development. Blood (2005) 106:1223–31. 10.1182/blood-2005-02-0551 PMC1895209 15860665

[108] Juban G Sakakini N Chagraoui H Cruz Hernandez D Cheng Q Soady K. Oncogenic Gata1 causes stage-specific megakaryocyte differentiation delay. Haematologica (2020). 10.3324/haematol.2019.244541 PMC8018159 32527952

[109] Nishinaka-Arai Y Niwa A Matsuo S Kazuki Y Yakura Y Hiroma T. Down syndrome-related transient abnormal myelopoiesis is attributed to a specific erythro-megakaryocytic subpopulation with GATA1 mutation. Haematologica (2020) 106:635–40. 10.3324/haematol.2019.242693 PMC7849752 32354872

[110] Chou S Lodish HF. Fetal liver hepatic progenitors are supportive stromal cells for hematopoietic stem cells. Proc Natl Acad Sci U S A (2010) 107:7799–804. 10.1073/pnas.1003586107 PMC2867886 20385801

[111] Zhang CC Lodish HF. Insulin-like growth factor 2 expressed in a novel fetal liver cell population is a growth factor for hematopoietic stem cells. Blood (2004) 103:2513–21. 10.1182/blood-2003-08-2955 14592820

[112] Maroz A Stachorski L Emmrich S Reinhardt K Xu J Shao Z. GATA1s induces hyperproliferation of eosinophil precursors in Down syndrome transient leukemia. Leukemia (2014) 28:1259–70. 10.1038/leu.2013.373 PMC4047213 24336126

[113] Wilson NK Foster SD Wang X Knezevic K Schütte J Kaimakis P. Combinatorial transcriptional control in blood stem/progenitor cells: genome-wide analysis of ten major transcriptional regulators. Cell Stem Cell (2010) 7:532–44. 10.1016/j.stem.2010.07.016 20887958

[114] Roy A Roberts I Norton A Vyas P. Acute megakaryoblastic leukaemia (AMKL) and transient myeloproliferative disorder (TMD) in Down syndrome: A multi-step model of myeloid leukaemogenesis. Br J Haematol (2009) 147:3–12. 10.1111/j.1365-2141.2009.07789.x 19594743

[115] Langebrake C Creutzig U Reinhardt D. Immunophenotype of Down syndrome acute myeloid leukemia and transient myeloproliferative disease differs significantly from other diseases with morphologically identical or similar blasts. Klin Padiatr (2005) 217:126–34. 10.1055/s-2005-836510 15858703

[116] Gamis AS Alonzo TA Gerbing RB Hilden JM Sorrell AD Sharma M. Natural history of transient myeloproliferative disorder clinically diagnosed in Down syndrome neonates: A report from the Children’s Oncology Group Study A2971. Blood (2011) 118:6752–9. 10.1182/blood-2011-04-350017 PMC3245202 21849481

[117] Zipursky A Brown EJ Christensen H Doyle J. Transient myeloproliferative disorder (transient leukemia) and hematologic manifestations of Down syndrome. Clin Lab Med (1999) 19:157–67. 10403079

[118] Homans AC Verissimo AM Vlacha V. Transient abnormal myelopoiesis of infancy associated with trisomy 21. Am J Pediatr Hematol Oncol (1993) 15:392–9. 8214361

[119] Massey GV Zipursky A Chang MN Doyle JJ Nasim S Taub JW. A prospective study of the natural history of transient leukemia (TL) in neonates with Down syndrome (DS): Children’s Oncology Group (COG) study POG-9481. Blood (2006) 107:4606–13. 10.1182/blood-2005-06-2448 16469874

[120] Muramatsu H Kato K Watanabe N Matsumoto K Nakamura T Horikoshi Y. Risk factors for early death in neonates with Down syndrome and transient leukaemia. Br J Haematol (2008) 142:610–5. 10.1111/j.1365-2141.2008.07231.x 18510680

[121] Watanabe K. Recent advances in the understanding of transient abnormal myelopoiesis in Down syndrome. Pediatr Int (2019) 61:222–9. 10.1111/ped.13776 30593694

[122] Hojo S Tsukimori K Kitade S Nakanami N Hikino S Hara T. Prenatal sonographic findings and hematological abnormalities in fetuses with transient abnormal myelopoiesis with Down syndrome. Prenat Diagn (2007) 27:507–11. 10.1002/pd.1718 17345586

[123] Strobelt N Ghidini A Locatelli A Vergani P Mariani S Biondi A. Intrauterine diagnosis and management of transient myeloproliferative disorder. Am J Perinatol (1995) 12:132–4. 10.1055/s-2007-994424 7779196

[124] Zipursky A Rose T Skidmore M Thorner P Doyle J. Hydrops fetalis and neonatal leukemia in Down syndrome. Pediatr Hematol Oncol (1996) 13:81–7. 10.3109/08880019609033374 8718505

[125] Federmann B Fasan A Kagan KO Haen S Fend F. Transient abnormal myelopoiesis/acute megakaryoblastic leukemia diagnosed in the placenta of a stillborn Down syndrome fetus with targeted next-generation sequencing. Leukemia (2015) 29:232–3. 10.1038/leu.2014.258 25179734

[126] Heald B Hilden JM Zbuk K Norton A Vyas P Theil KS. Severe TMD/AMKL with GATA1 mutation in a stillborn fetus with Down syndrome. Nat Clin Pract Oncol (2007) 4:433–8. 10.1038/ncponc0876 17597708

[127] Ishigaki H Miyauchi J Yokoe A Nakayama M Yanagi T Taga T. Expression of megakaryocytic and myeloid markers in blasts of transient abnormal myelopoiesis in a stillbirth with Down syndrome: report of histopathological findings of an autopsy case. Hum Pathol (2011) 42:141–5. 10.1016/j.humpath.2010.06.012 20970166

[128] Muramatsu H Watanabe T Hasegawa D Myoung-ja P Iwamoto S Taga T. Prospective Study of 168 Infants with Transient Abnormal Myelopoiesis with Down Syndrome: Japan Pediatric Leukemia/Lymphoma Study Group, TAM-10 Study. Blood (2015) 126:1311. 10.1182/blood.V126.23.1311.1311

[129] Flasinski M Scheibke K Zimmermann M Creutzig U Reinhardt K Verwer F. Low-dose cytarabine to prevent myeloid leukemia in children with Down syndrome: TMD Prevention 2007 study. Blood Adv (2018) 2:1532–40. 10.1182/bloodadvances.2018018945 PMC6039662 29959152

[130] Isaacs H. Fetal and neonatal leukemia. J Pediatr Hematol Oncol (2003) 25:348–61. 10.1097/00043426-200305000-00002 12759620

[131] Malinge S Ragu C Della-Valle V Pisani D Constantinescu SN Perez C. Activating mutations in human acute megakaryoblastic leukemia. Blood (2008) 112:4220–6. 10.1182/blood-2008-01-136366 18755984

[132] Kiyoi H Yamaji S Kojima S Naoe T. JAK3 mutations occur in acute megakaryoblastic leukemia both in Down syndrome children and non-Down syndrome adults. Leukemia (2007) 21:574–6. 10.1038/sj.leu.2404527 17252020

[133] Walters DK Mercher T Gu T-L O’Hare T Tyner JW Loriaux M. Activating alleles of JAK3 in acute megakaryoblastic leukemia. Cancer Cell (2006) 10:65–75. 10.1016/j.ccr.2006.06.002 16843266

[134] Klusmann JH Reinhardt D Hasle H Kaspers GJ Creutzig U Hahlen K. Janus kinase mutations in the development of acute megakaryoblastic leukemia in children with and without Down’s syndrome. Leukemia (2007) 21:1584–7. 10.1038/sj.leu.2404694 17443226

[135] Waldman T. Emerging themes in cohesin cancer biology. Nat Rev Cancer (2020) 20:504–15. 10.1038/s41568-020-0270-1 32514055

[136] Thota S Viny AD Makishima H Spitzer B Radivoyevitch T Przychodzen B. Genetic alterations of the cohesin complex genes in myeloid malignancies. Blood (2014) 124:1790–8. 10.1182/blood-2014-04-567057 PMC4162108 25006131

[137] Garnett C Cruz Hernandez D Vyas P. GATA1 and cooperating mutations in myeloid leukaemia of Down syndrome. IUBMB Life (2020) 72:119–30. 10.1002/iub.2197 31769932

[138] Mazumdar C Shen Y Xavy S Zhao F Reinisch A Li R. Leukemia-Associated Cohesin Mutants Dominantly Enforce Stem Cell Programs and Impair Human Hematopoietic Progenitor Differentiation. Cell Stem Cell (2015) 17:675–88. 10.1016/j.stem.2015.09.017 PMC4671831 26607380

[139] Mullenders J Aranda-Orgilles B Lhoumaud P Keller M Pae J Wang K. Cohesin loss alters adult hematopoietic stem cell homeostasis, leading to myeloproliferative neoplasms. J Exp Med (2015) 212:1833–50. 10.1084/jem.20151323 PMC4612095 26438359

[140] Horsfield JA Anagnostou SH Hu JK-H Cho KH Geisler R Lieschke G. Cohesin-dependent regulation of Runx genes. Dev (Cambridge Engl) (2007) 134:2639–49. 10.1242/dev.002485 17567667

[141] Viny AD Ott CJ Spitzer B Rivas M Meydan C Papalexi E. Dose-dependent role of the cohesin complex in normal and malignant hematopoiesis. J Exp Med (2015) 212:1819–32. 10.1084/jem.20151317 PMC4612085 26438361

[142] Mercher T Wernig G Moore SA Levine RL Gu T-L Fröhling S. JAK2T875N is a novel activating mutation that results in myeloproliferative disease with features of megakaryoblastic leukemia in a murine bone marrow transplantation model. Blood (2006) 108:2770–9. 10.1182/blood-2006-04-014712 PMC1895587 16804112

[143] de Vita S Mulligan C McElwaine S Dagna-Bricarelli F Spinelli M Basso G. Loss-of-function JAK3 mutations in TMD and AMKL of Down syndrome. Br J Haematol (2007) 137:337–41. 10.1111/j.1365-2141.2007.06574.x 17456055

[144] Varghese L Levy G Constantinescu SN. Thrombopoietin receptor activation by a Down syndrome myeloid leukemia variant of the common beta chain of the IL3, IL5 and GM-CSF signalling complexes. HemaSphere (2020) 4:294943; S123.

[145] Malinge S Chlon T Doré LC Ketterling RP Tallman MS Paietta E. Development of acute megakaryoblastic leukemia in Down syndrome is associated with sequential epigenetic changes. Blood (2013) 122:e33–43. 10.1182/blood-2013-05-503011 PMC3790517 23980066

[146] Shaham L Vendramini E Ge Y Goren Y Birger Y Tijssen MR. MicroRNA-486-5p is an erythroid oncomiR of the myeloid leukemias of Down syndrome. Blood (2015) 125:1292–301. 10.1182/blood-2014-06-581892 PMC4335082 25533034

[147] Schwarzer A Emmrich S Schmidt F Beck D Ng M Reimer C. The non-coding RNA landscape of human hematopoiesis and leukemia. Nat Commun (2017) 8:218. 10.1038/s41467-017-00212-4 28794406 PMC5550424

[148] Emmrich S Streltsov A Schmidt F Thangapandi VR Reinhardt D Klusmann J-H. LincRNAs MONC and MIR100HG act as oncogenes in acute megakaryoblastic leukemia. Mol Cancer (2014) 13:171. 10.1186/1476-4598-13-171 25027842 PMC4118279

[149] Saida S Watanabe K Sato-Otsubo A Terui K Yoshida K Okuno Y. Clonal selection in xenografted TAM recapitulates the evolutionary process of myeloid leukemia in Down syndrome. Blood (2013) 121:4377–87. 10.1182/blood-2012-12-474387 23482930

[150] Lopez CK Noguera E Stavropoulou V Robert E Aid Z Ballerini P. Ontogenic Changes in Hematopoietic Hierarchy Determine Pediatric Specificity and Disease Phenotype in Fusion Oncogene-Driven Myeloid Leukemia. Cancer Discov (2019) 9:1736–53. 10.1158/2159-8290.CD-18-1463 31662298

[151] Bertuccio SN Boudia F Cambot M Lopez CK Lordier L Donada A. The Pediatric Acute Leukemia Fusion Oncogene ETO2-GLIS2 Increases Self-Renewal and Alters Differentiation in a Human Induced Pluripotent Stem Cells-Derived Model. HemaSphere (2020) 4:e319. 10.1097/HS9.0000000000000319 32072139 PMC7000481

[152] Cardin S Bilodeau M Roussy M Aubert L Milan T Jouan L. Human models of NUP98-KDM5A megakaryocytic leukemia in mice contribute to uncovering new biomarkers and therapeutic vulnerabilities. Blood Adv (2019) 3:3307–21. 10.1182/bloodadvances.2019030981 PMC6855103 31698461

